# Robotic-assisted radical prostatectomy learning curve for experienced laparoscopic surgeons: does it really exist?

**DOI:** 10.1590/S1677-5538.IBJU.2014.0485

**Published:** 2016

**Authors:** Marcos Tobias-Machado, Anuar Ibrahim Mitre, Mauricio Rubinstein, Eduardo Fernandes da Costa, Alexandre Kyoshi Hidaka

**Affiliations:** 1 Divisão de Urologia, Faculdade de Medicina do ABC, Santo André, SP, Brasil;; 2 Divisão de Urologia, Jundiaí Faculdade de Medicina de Jundiaí, SP, Brasil;; 3 Hospital Sírio-Libanês, SP, Brasil;; 4 Divisão de Urologia, Seção de Cirurgia Minimamente Invasiva do Hospital Universitário Gaffrée e Guinle (UNIRIO), Rio de Janeiro, Brasil;; 5 Faculdade de Medicina do ABC, Santo André, SP, Brasil

**Keywords:** Laparoscopy, Prostatectomy, Prostatic Neoplasms, Robotic Surgical Procedures

## Abstract

**Background:**

Robotic-assisted radical prostatectomy (RALP) is a minimally invasive procedure that could have a reduced learning curve for unfamiliar laparoscopic surgeon. However, there are no consensuses regarding the impact of previous laparoscopic experience on the learning curve of RALP. We report on a functional and perioperative outcome comparison between our initial 60 cases of RALP and last 60 cases of laparoscopic radical prostatectomy (LRP), performed by three experienced laparoscopic surgeons with a 200+LRP cases experience.

**Materials and Methods:**

Between January 2010 and September 2013, a total of 60 consecutive patients who have undergone RALP were prospectively evaluated and compared to the last 60 cases of LRP. Data included demographic data, operative duration, blood loss, transfusion rate, positive surgical margins, hospital stay, complications and potency and continence rates.

**Results:**

The mean operative time and blood loss were higher in RALP (236 versus 153 minutes, p<0.001 and 245.6 versus 202ml p<0.001). Potency rates at 6 months were higher in RALP (70% versus 50% p=0.02). Positive surgical margins were also higher in RALP (31.6% versus 12.5%, p=0.01). Continence rates at 6 months were similar (93.3% versus 89.3% p=0.43). Patient’s age, complication rates and length of hospital stay were similar for both groups.

**Conclusions:**

Experienced laparoscopic surgeons (ELS) present a learning curve for RALP only demonstrated by longer operative time and clinically insignificant blood loss. Our initial results demonstrated similar perioperative and functional outcomes for both approaches. ELS were able to achieve satisfactory oncological and functional results during the learning curve period for RALP.

## INTRODUCTION

Prostate cancer is the most common non-cutaneous men malignancy and the second leading cause of cancer related mortality in Brazil (1). Minimally invasive approaches for prostate cancer have evolved significantly after 2000.

Laparoscopic radical prostatectomy (LRP) demonstrated improved visualization of the pelvic anatomy, improvements in potency and urinary rates, lower blood loss, while upholding principles of oncological therapy (2-6). Although, this technique presented a limited expansion due to the steep learning curve, which requires at least 60 cases to obtain proficiency (6).

Recently, robot-assisted radical prostatectomy (RALP) brought several mechanisms which may significantly decrease the learning curve for unfamiliar laparoscopically surgeons (2). The Da Vinci surgical system (Intuitive Surgical, Sunnyvale, California, USA) magnification, robotic-wrist instrumentation and increased degrees of freedom, associated with the 3-dimensional visualization provided surgeons extremely detailed pelvic anatomy which enables the appropriate prostate extirpation (7-9). This minimally invasive technique has received widespread acceptance by physicians and patients and was established as the standard surgical treatment for localized prostate cancer in the US (10-12).

In Brazil, the Da Vinci System was introduced in 2008. However, it was implemented only in 9 hospital centers (Albert Einstein, Sirio Libanes, Oswaldo Cruz, Nove de Julho, INCA, Samaritano, HC Porto Alegre, ICESP and Fundação Pio XII). In addition, this high-cost technology is not provided by health insurances, being mostly performed by private services, which provides low volume of RALP for most urologists.

The aim of this study was to report our initial experience and assess the learning curve of experienced laparoscopic surgeons in robot-assisted radical prostatectomy (RALP). We compared perioperative, functional and oncological outcomes between RALP and LRP.

## MATERIALS AND METHODS

The project was approved by the Ethics Committee for Analysis of Research Projects of the involved institutions.

A retrospective review of prospectively collected data was performed from 2008 to 2013, including 120 patients with localized low or intermediate risk of prostate cancer who were indicated for surgical treatment. All selected cases presented previous urinary and potency rates preserved. Patients with previous prostate cancer treatment, neoadjuvant or adjuvant hormonal treatment were excluded from the study. The robotic procedures were performed at a private hospital while the LRP in public and private hospitals.

Preoperative, perioperative, oncological and functional outcomes of the first 60 cases of robot-assisted radical prostatectomy were compared to the last 60 consecutive cases of laparoscopic radical prostatectomy. All procedures were performed by three experienced surgeons with a 200+experience in LRP, under the same defined protocol.

Data included demographic characteristics, operative parameters (operative time, blood loss, positive surgical margin, complications, conversion and transfusion rates and postoperative (early urinary and potency continence and post-operative stay).

## SURGICAL TECHNIQUE

### Robotic-assisted laparoscopic radical prostatectomy

The RALP was performed using the S and Si da Vinci Robotic System (Intuitive Surgical, Sunnyvale, CA). First, the patient was positioned supine in low lithotomy in a 15º Trendelenburg position. All cases were performed transperitoneally using the six-port technique as described by Patel et al. (13). Non robotic ports were placed higher or above umbilicus’s level in order to provide maximum range of motion to the assistant. Dorsal venous complex was initially isolated and ligated. The seminal vesicles dissection was then performed and prostatic pedicles ligation was carried out. Nerve-sparing surgery was performed when using a clip technique without the use of any kind of thermal energy. Finally, the running vesicourethral anastomosis was performed as described by Van Velthoven et al. with conventional 3-0 barbed sutures.

### Laparoscopic radical prostatectomy

Pure laparoscopic cases were performed with five-port extraperitoneal approach described by us previously (14, 15). The patient was placed in supine position with Y-shaped abduction of lower limbs. Optics trocar was inserted in the umbilical incision, two trocars were inserted in the pararectal external area and two in the iliac fossa. Vascular control of dorsal venous complex was performed using a 2-0 polygalactine suture with CT-1. The bladder neck was incised and the vasa deferentia and seminal vesicles were dissected. Posterior prostate pedicles were clipped and incised. The dorsal vein complex and urethra were incised and the prostate released. Continuous 3-0 monocryl or 3-0 barbed sutures were used to perform the vesicourethral Van Velthoven anastomosis.

### Statistical analysis

The statistical analyses were performed using SPSS software (IBM® SPSS® Statistics20; SPSS, Inc., Chicago, IL, USA). The significance level was defined as 0.05 (5%). All confidence intervals used in this study were constructed with a 95% confidence level.

The paired Student t test was used to assess quantitative data and compare means (age, operative time, blood loss, PSA level). The two-samples z test was used to compare intraoperative complications, continence and potency rates, positive surgical margins, transfusion rate, Gleason score, pathologic stage and nerve sparring between the groups.

## RESULTS

Patients who have undergone LRP and RALP were similar in terms of age and ranged from 50 to 70 (p=0.99). PSA level, Gleason score and pathologic stage (T2, T3) were also similar between the groups (Table-1). Bilateral nerve sparing was performed in 83.3% in RALP and 73.3% in LRP and both were considered similar (p=0.18).

**Table 1:** Preoperative patient characteristics. The groups were similar in terms of age, PSA level, Gleason grade, nerve sparing and pathologic stage (T2, T3).

	LRP	RALP	P value
	**n=60**	**n=60**	
		
Age (range, SD) years	60.56±11.6	60.58±7.94	0.99
PSA (range, SD) ng/mL	7.05±3.70	6.17±2.63	0.13
**Gleason grade (%)**			
**≤6**	46.25%	46.6%	0.96
**7**	35.00%	45.0%	0.26
**>7**	18.75%	8.4%	0.09
**Pathologic stage (%)**			
**pT2**	87.20%	81.6%	0.74
**pT** 3	12.80%	18.4%	0.4
**Nerve Sparing (%)**			
Unilateral	26.7%	16.7%	0.18
Bilateral	73.3%	83.3%	0.18

Mean operative time was longer in RALP (236.1±42.95) compared to LRP (153.51±41.8 p<0.001). A significantly difference was found in the blood loss (245.6±33.71 versus 202±73.3 p<0.001). Complications occurred in 10.3% of patients who underwent LRP and 6.6% in RALP. Visceral and rectal injuries, blood transfusion, wound infection, urinary tract infection and retention were included. No conversion to open or laparoscopic surgery was performed (Table-2). The length of hospital stay was similar between the groups (p=0.92) and ranged from 1-3 days.

**Table 2:** Perioperative outcomes. Robotic-assisted radical prostatectomy presented longer operative time and higher blood loss when compared to LRP.

	LRP	RALP	P value
	**n=60**	**n=60**	
		
**Operative time (minutes)**	153.51±41.8	236.1±42.95	<0.001
**Blood loss (ml)**	202±73.3	245.6±33.71	<0.001
**Intraoperative complications (%)**	10.30%	6.6%	0.46
**Hospital stay (days)**	1.38	1.60	0.92
**Transfusion rate (%)**	0%	0%	1.0

Functional and oncological outcomes are described in Table-3. Continence rates at six months was higher in RALP (70% versus 50% p=0.02). Potency rates at six months were similar (93.3% versus 89.3% p=0.43). Positive surgical margins was higher in RALP when compared to LRP (31.6% versus 12.5% p=0.01).

**Table 3:** Functional and oncological outcomes. Robotic-assisted radical prostatectomy presented higher percentage of potency continence at six months and positive surgical margins.

	LRP	RALP	P value
	**n=60**	**n=60**	
		
Continence rates at six month (%)	89.3%	93.3%	0.43
Potency rates at six month (%)	50%	70%	0.02
Positive Surgical Margins (%)	12.5%	21.6%	0.18
pT2	8%	12.5%	0.7
pT3	33%	50%	0.1

## DISCUSSION

Laparoscopic radical prostatectomy was the first successful minimally invasive procedure that provided several benefits concerning potency and urinary continence, blood loss, while upholding principles of oncological therapy (2). However, the two-dimensional image associated with lower range of motion turned LRP into a challenging procedure, which presents a steep learning curve that requires nearly 70 cases to attain proficiency (6, 15).

Robotic assisted radical prostatectomy emerged as an effective alternative to LRP. The Da Vinci 3-dimensional image, magnification, multi-joints devices, increased degrees of freedom significantly improved surgical ergonomics and therefore decreased the learning curve of LRP. RALP has received worldwide acceptance by urologists and is on the verge of becoming the preferred surgical treatment of localized prostate cancer (12, 16-18).

However, the high cost of this technology remains as the primary obstacle towards RALP expansion. The Da Vinci system is evaluated at 2 million euros and its maintenance increases financial burden by $2.698 per patient given an average of 126 cases per year. Previous reports estimated that a total of 75 cases per year with an average operation time of three hours per case are necessary to be cost-effective in the United States (16, 19). In Brazil, this system was introduced in 2008 and was implemented only in 9 hospital centers. INCA’s hospital (Instituto Nacional do Câncer) and ICESP (Instituto do Câncer do Estado de São Paulo) were the first public services that provided the Da Vinci System in Brazil. Therefore, based on the medical system without a reference system of patient’s, a low volume of procedures is performed by several urologists who are familiar with this technology. So, this condition may justify the few reports about the current situation of RALP in Brazil.

To our knowledge, this is the first Brazilian series that analyzes the learning curve of experienced laparoscopic surgeons and compare perioperative and functional outcomes between RALP and LRP. In this preliminary report, we found differences and similarities between the groups outcomes.

RALP operative time was longer than LRP, which is in accordance with previous larger series which estimated a range from 140 to 354 min (8, 11, 20-22). Menon et al. reported in early series of RALP a progressive decrease of operative time over time which is not observed in LRP (23). This finding suggests that further experience could lead to similar operation time. Estimated blood loss was higher in RALP and is in accordance with previous reports which reported an average of 234ml with a range of 75-500ml (20-22). Estimated blood loss was higher in RALP (approximately 50ml), however it was clinically insignificant and blood transfusion was not necessary in any case. This difference could be explained by the longer operative time of RALP.

Robotic-assisted radical prostatectomy presents several potential complications. Some authors include catheterization time, symptomatic lymphocele, hematoma, emphysema whereas other uses the Clavien grading system for short-term complications (11, 21, 24). In our initial experience we presented the most common complications and our rate was 10%, in accordance with most reports (22, 24, 25). Both RALP and LRP present similar incidence of conversion to open surgery, which are significantly low (10). In our experience, no procedures needed conversion or transfusions. Length of hospital stay is usually associated with perioperative complications and patient’s well-being, and we found no differences between LRP and RALP.

Continence rate at six month was significantly equal between our groups (93.3% versus 89.3%). This finding will be definitive only after a one-year evaluation. Ficarra’s et al. meta-analysis observed that RALP was significantly superior to LRP in terms of 12-month urinary continence recovery. Although he concluded that the prevalence of urinary incontinence after RALP is influenced by several factors including preoperative patient characteristics, surgeon experience, surgical technique and collective methods, which hinder this assessment (7).

However, potency rates were higher in RALP when compared to LRP (70% versus 50%). This finding is in accordance with Ficarra’s et al. meta-analysis that demonstrated a significant advantage in favor of RALP in comparison with RRP in terms of 12-month potency rates (26). In addition, this finding suggests that further experience on RALP and longer follow-up could lead to early potency rates, even for experienced laparoscopic surgeons.

Positive surgical margin rates were significantly similar between the groups (21.6% for RALP and 12.5% in LRP). This finding was similar to previous studies which RALP ranged from 12.3% to 17.2% and LRP 11-29%. Most series reported no statistically significant difference between LRP and RALP (16, 20, 23, 27).

Currently, there is no consensus over the superiority of RALP or LRP in the treatment of localized prostate cancer. Several studies compared both techniques and presented different results rather in favor of RALP or LRP (2, 11, 16, 19, 27-30). We believe that the Da Vinci System is a technological evolution which provides more detailed information regarding this complex procedure. On the other hand, considering the low volume of Da Vinci’s system installed in Brazil during the 7 last years, most urologists won’t have access to robotic surgery in Brazil for a long time, which turns LRP into a feasible alternative. Additionally, LRP may be a shortcut for reducing the learning curve of RALP. We observed that surgeons who are proficient in LRP and have low volume of RALP presents a learning curve that did not jeopardize their oncological and functional outcomes. Similar to USA, where massive RALP expansion turned it to be the established surgical treatment for localized prostate cancer, it will be natural that RALP replace LRP in the future, when technology and trained surgeons could be largely available (10, 23, 27, 28).

In our study we observed that an experienced laparoscopic surgeon was able to attain perioperative and functional outcomes in his/her initial results similar to surgeons who present higher experience in RALP. The previous experience on LRP could decrease the learning curve of RALP, mainly concerning the similarity of surgical steps and pelvic anatomy visualization. Therefore, the learning curve would be mainly related to the management of the robotic system new features such as multi-joints devices and absence of tactile feedback.

We consider the limitations of our initial experience which was performed in a low volume center for both procedures in private hospitals. Our results aid the comparison between LRP and RALP for experienced laparoscopic surgeons, however our results should be considered indicative only. Longer oncologic and functional follow-up are still required.

Experienced laparoscopic surgeons present a learning curve when first performing an RALP, demonstrated only by longer operative time. Even though our perioperative and functional outcomes were similar for both approaches and in accordance with previous reports (11, 21, 31). ELS were able to achieve satisfactory oncological and functional results during the learning curve period for RALP.
